# Mechanical Enhancements of Electrospun Silica Microfibers with Boron Nitride Nanotubes

**DOI:** 10.3390/nano16010069

**Published:** 2026-01-03

**Authors:** Dingli Wang, Nasim Anjum, Zihan Liu, Changhong Ke

**Affiliations:** 1 Department of Mechanical Engineering, State University of New York at Binghamton, Binghamton, NY 13902, USA; 2 Materials Science and Engineering Program, State University of New York at Binghamton, Binghamton, NY 13902, USA

**Keywords:** boron nitride nanotube, ceramic matrix composite, electrospinning, mechanical enhancement, interfacial load transfer

## Abstract

We investigate the mechanical properties of electrospun boron nitride nanotube (BNNT)-reinforced silica nanocomposite microfibers. The incorporation of small amounts of BNNTs (0.1, 0.3, and 0.5 wt.%) into silica results in significant enhancements in the bulk mechanical performance, including up to a 26.4% increase in Young’s modulus, a 19.4% increase in tensile strength, and a 12.8% increase in toughness. These improvements are attributed to the excellent nanotube alignment achieved via electrospinning and the effective transfer of interfacial loads at the BNNT–silica interface. Micromechanical analysis based on in situ Raman measurements reveals that the maximum interfacial shear stress in the electrospun BNNT–silica microfiber reaches about 341 MPa. This study provides new insights into the process–structure–property relationship and reinforcement mechanisms in nanotube-reinforced ceramic nanocomposites, thereby advancing the development of lightweight, strong, tough, and durable ceramic materials.

## 1. Introduction

The incorporation of nanofibers into ceramics offers a promising approach to addressing the long-standing challenge posed by the intrinsic brittleness of ceramics. The combination of lightweight properties and high strength, toughness, and durability in nanofiber-reinforced ceramic nanocomposites makes them highly attractive for a broad range of industrial applications, including aerospace and electronic components [1]. Among various fillers, boron nitride nanotubes (BNNTs) have recently emerged as one of the most promising reinforcements for ceramics owing to their extraordinary structural and physical properties and thermal stability. Structurally, BNNTs are one-dimensional, hollow tubular nanostructures composed of a hexagonal honeycomb network of alternating boron and nitrogen atoms. This architecture imparts exceptional stiffness and strength to BNNTs at very low density. BNNTs exhibit a Young’s modulus of up to 1.3 TPa and a tensile strength of up to 60 GPa [2,3,4], rivaling those of their pure carbon counterparts, carbon nanotubes (CNTs). In addition to their mechanical robustness, BNNTs demonstrate superior thermal stability, maintaining structural integrity up to 900 °C in air [5] and over 1800 °C in inert atmospheres [6], whereas CNTs begin to oxidize at approximately 450 °C. A particularly unique feature of BNNTs is their simultaneous high thermal conductivity (~3000 W/m K) [7] and large electronic bandgap (5–6 eV) [8], rendering them excellent thermal conductors yet electrical insulators. When incorporated into ceramic matrices, BNNTs enhance heat dissipation while preserving electrical insulation and low-density properties highly desirable for aerospace, electronics, and thermal management applications. Moreover, BNNTs possess neutron radiation-shielding capabilities [9], further enhancing their suitability for extreme environments, such as spacecraft and nuclear systems. The partially ionic and covalent nature of the B–N bond results in asymmetric charge distribution and anisotropic interfacial energy landscapes, promoting strong interfacial shear resistance at the BNNT–ceramic interface and dissipating energy through frictional sliding during nanotube pullout [10,11]. The strong binding interactions between BNNTs and ceramics enable the embedded nanotubes to act as crack stoppers, ultimately yielding enhanced fracture resistance. Collectively, these exceptional structural, mechanical, and multifunctional characteristics make BNNTs highly effective fillers for enhancing both the mechanical integrity and functional performance of ceramics.

BNNTs have been shown to effectively reinforce and toughen a wide variety of ceramic matrices, including both conventional ceramics (e.g., silica and alumina) and polymer-derived ceramics. For example, earlier studies have shown that incorporating 5 wt.% BNNTs increased the flexural strength and fracture toughness of silica by 131% and 109%, respectively [12], and enhanced the elastic modulus and fracture toughness of alumina by 35% and 51%, respectively [13]. More recent studies have demonstrated that substantial mechanical enhancements can be achieved at much lower BNNT loadings. Tank et al. found that adding only 0.1 wt.% BNNTs produced increases of 72% in bending modulus and 55% in bending strength in silica [14]. Likewise, Nasim et al. showed that incorporating 0.5 wt.% BNNTs into silica resulted in increases in bending strength by 153% and fracture toughness by 167% [15]. Similar enhancements have been reported for alumina, where the addition of 0.6 wt.% BNNTs in alumina yielded improvements of 71% in flexural strength and 74% in fracture toughness [16]. In polymer-derived silicon oxycarbide (SiOC), the introduction of 1 wt.% BNNTs led to a 150% increase in flexural strength and a 230% increase in fracture toughness [17].

Effective interfacial load transfer between nanotubes and ceramic matrices is widely recognized as a key mechanism governing the remarkable improvements in the bulk mechanical performance of nanotube-reinforced ceramics. The efficiency of this load transfer determines the extent to which the exceptional stiffness and strength of individual BNNTs can be harnessed at the macroscopic level. Strong interfacial binding enables effective transmission of external stresses from the ceramic matrix to the nanotubes, thereby enhancing the composite’s strength, stiffness, and toughness. The quality of interfacial load transfer is highly sensitive to nanotube separation, dispersion, and alignment within the ceramic matrix. Nanotube alignment is particularly critical, as the ability of nanotubes to bear and redistribute applied stresses depends on their orientation relative to the direction of loading. Across many BNNT–ceramic systems prepared via either conventional molding [12,15,17] or additive manufacturing [14,16,18,19], the nanotubes are generally embedded with random orientations. This random arrangement significantly diminishes the effective load-bearing capacity and reduces the achievable mechanical enhancements. Therefore, controlling nanotube alignment during processing is essential to maximize reinforcement efficiency and realize the full potential of BNNTs as nanoscale reinforcements.

In this study, we employ an electrospinning-based fabrication approach to produce BNNT-reinforced silica nanocomposite microfibers and quantitatively investigate both their local interfacial behavior and bulk mechanical properties. The electrospinning process results in excellent alignment of BNNTs along the microfiber’s longitudinal axis. This alignment arises from frictional drag between the nanotubes and the highly viscous jet under strong electrostatic fields, which orients the nanotubes along the fiber elongation direction during the spinning process [20,21]. Such controlled alignment enables efficient stress transfer across the BNNT–silica interface, resulting in a maximum interfacial shear stress of approximately 341 MPa—a value substantially higher than previously reported for bulk BNNT–silica systems with randomly oriented nanotubes. The enhanced alignment and interfacial binding collectively lead to significant improvements in bulk mechanical performance, including higher stiffness, strength, and toughness. These findings highlight the crucial role of processing-induced nanotube orientation and interfacial load transfer in determining the macroscopic mechanical behavior of ceramic nanocomposites. To the best of our knowledge, this is the first report on the mechanical reinforcement of electrospun ceramic fibers with BNNTs. By establishing a clear correlation between manufacturing protocols, microstructural alignment, and mechanical performance, this work provides new insights into the process–structure–property relationship governing the bulk mechanical properties of nanotube-reinforced ceramics. The results contribute to the rational design and manufacturing of next-generation ceramic materials that are lightweight, strong, tough, and durable, suitable for demanding structural and multifunctional applications.

## 2. Materials and Methods

### 2.1. Sample Preparation

The BNNTs used in this study (BNNT Materials, Newport News, VA, USA) were synthesized using high-temperature–pressure (HTP) methods [22]. HTP-BNNTs are predominantly double-walled nanotubes with a median diameter of 2.9 nm [23]. Recent studies report that the as-received BNNTs have hexagonal boron nitride (hBN) purity exceeding 99% and a BNNT purity of approximately 88% [15]. The silica precursor, tetraethyl orthosilicate (TEOS, purity ≥ 99.0%), and the polyethylene oxide (PEO, molecular weight = 600,000 g mol^−1^) were obtained from Sigma-Aldrich Co. (St. Louis, MO, USA). Hydrochloric acid (HCl, ~37.5%) aqueous solution and acetone (purity ≥ 99.5%) were supplied by Fisher Scientific Co. (Waltham, MA, USA) and VWR International Ltd. (Radnor, PA, USA), respectively.

The as-received BNNTs were first dispersed in a mixed solvent of acetone and HCl using an ultrasonication bath (155 W, 42 kHz) for one hour to achieve uniform dispersion. The length of ultrasonication-dispersed BNNTs typically ranges from several hundred nanometers to tens of micrometers [5]. The silica precursor solution was prepared by mixing TEOS with 4 wt.% PEO at 50 °C under continuous magnetic stirring until a viscous, homogeneous solution was obtained. The molar ratio of components in the electrospinning solution was TEOS:H_2_O:acetone:HCl = 1:4.6:2.2:0.06. Electrospinning was conducted using a custom-built setup equipped with a 30 kV power supply (setting 6 kV), a digitally controlled syringe pump, and a 22-gauge stainless-steel needle (inner diameter ~413 μm). The electrospun silica microfibers were collected on grounded aluminum foils positioned 10 cm from the needle tip at a flow rate of 3 mL/h under room temperature (~21 °C) and 30% relative humidity. The collected microfibers were dried in air for one hour and subsequently calcined in air at 800 °C for 2 h in a box furnace (Thermcraft Inc., Winston-Salem, NC, USA) at a heating rate of 1 °C/min. Electrospun silica microfibers containing BNNT concentrations of up to 0.5 wt.% (weight percent, unless otherwise specified) were successfully fabricated using this method.

### 2.2. Characterization

Fourier transform infrared (FTIR) spectroscopy was performed using a Nicolet 8700 spectrometer in attenuated total reflection (ATR) mode. X-ray diffraction (XRD) patterns were collected using a Cu-sealed tube (Cu Kα radiation, λ = 1.54 Å) operating at 45 kV and 40 mA. Thermogravimetric analysis (TGA) was conducted in air on a Q50 analyzer (TA Instruments, New Castle, DE, USA) at a heating rate of 10 °C/min from room temperature to 800 °C to evaluate the kinetics of weight loss. The bulk mechanical properties of pure silica and BNNT–silica microfibers were evaluated via tensile testing in accordance with ASTM C1557, using six specimens tested for each BNNT loading. Mechanical tests were performed using an ADMET micro-mechanical tester equipped with a 5 N load cell, operating at a crosshead displacement rate of 1 mm/min. Before testing, individual microfibers with diameters of 10–20 μm were selected under a Nikon optical microscope (see Supplementary Figure S1 in the Supplementary Material). Each tested microfiber was firmly glued to a custom-built testing frame, which was clamped to the tester’s grips. A gauge length of 20 mm was employed. Toughness was determined by integrating the area under the stress–strain curve up to fracture. The microstructure and morphology of the samples were examined using a field-emission scanning electron microscope (FE-SEM, Supra 55, Zeiss, Jena, Germany). The microfiber density was measured using a densitometer (YDK03, Sartorius, Göttingen, Germany) based on the Archimedean principle. Raman spectroscopy, including polarized Raman measurements, was carried out using a Renishaw InVia Raman microscope (Renishaw PLC, Gloucestershire, UK) equipped with a 532 nm excitation laser, a 2400 lines·mm^−1^ grating, and a 50× objective lens. In situ micromechanical Raman spectroscopy was performed by acquiring spectra at each 0.1% strain increment using a TST350 micromechanical tester (Linkam Scientific Instruments, Redhill, UK) integrated with a Renishaw Raman system.

## 3. Results and Discussion

### 3.1. Structural and Material Properties

**Figure 1:**
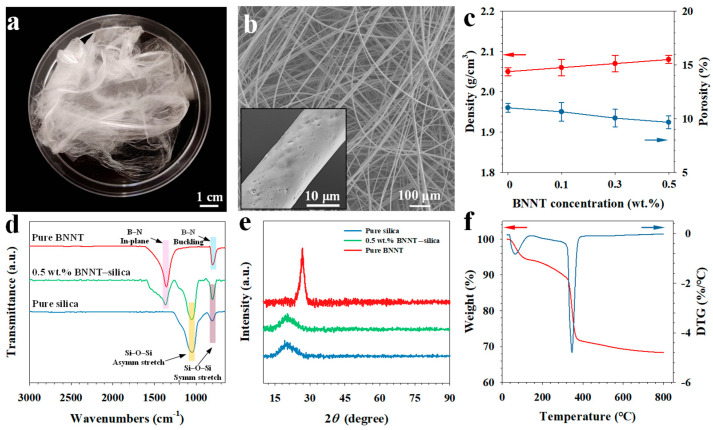
Structural and material characterization of electrospun BNNT–silica nanocomposite microfibers. (**a**) Optical image and (**b**) SEM image of 0.5 wt.% BNNT–silica microfiber meshes (inset: enlarged view of a single microfiber showing surface pits). (**c**) Density and porosity. (**d**) FTIR spectra and (**e**) XRD patterns of pure silica microfibers, 0.5 wt.% BNNT–silica microfibers, and pure BNNTs. (**f**) Thermogravimetric and corresponding differential thermogravimetric curves of 0.5 wt.% BNNT–silica microfibers.

Figure 1a shows a representative optical image of as-electrospun 0.5 wt.% BNNT–silica microfiber meshes, and Figure 1b shows the structural morphology of individual BNNT–silica microfibers. The silica microfibers are formed by hydrolyzing TEOS, followed by condensation reactions that yield the final silica network. The chemical transformation of TEOS to silica is expressed as follows [24]:

(1) $$\mathrm{Si}{(OC_{2}H_{5})}_{4}+2H_{2}O\;\to \;{\mathrm{Si}O}_{2}+4C_{2}H_{5}\mathrm{OH}$$

The diameter of electrospun microfibers can be controlled by adjusting processing parameters [25], such as the applied voltage and solution flow rate, enabling the fabrication of uniform fibers with diameters ranging from several hundred nanometers to tens of micrometers. The microfiber surfaces are generally smooth with a sparse distribution of small surface pits, as shown in the inset of Figure 1b.

The electrospun BNNT–silica microfibers possess porous structures. Figure 1c presents the measured bulk density and corresponding porosity, showing a moderate increase in fiber density with increasing BNNT content. The average density of the pristine fibrous silica is approximately 2.05 g/cm^3^, which is noticeably lower than the theoretical density of amorphous silica (2.3 g/cm^3^ [26]). Incorporating 0.1–0.5 wt.% BNNTs slightly raises the density to 2.06–2.08 g/cm^3^, with the corresponding porosity decreasing from about 11.0% to 9.7%. This trend can be attributed to BNNTs’ propensity to promote local microstructural ordering and enhance the crystallinity of the amorphous ceramic matrix [17], thereby increasing the composite’s overall density.

Figure 1d presents the FTIR spectra of BNNTs, pure silica microfibers, and 0.5 wt.% BNNT–silica microfibers, highlighting their characteristic vibrational features. The pure silica microfibers exhibit prominent absorption bands at 1050 cm^−1^ and 800 cm^−1^, which correspond to the asymmetric and symmetric stretching vibrations of the Si–O–Si bonds, respectively [27]. BNNTs display two characteristic peaks at approximately 801 cm^−1^ and 1355 cm^−1^, which are attributed to the out-of-plane buckling and in-plane stretching vibrations of the B–N bond, respectively [28]. The pronounced out-of-plane vibration observed near 800 cm^−1^ further indicates the high structural purity of the employed BNNTs. Importantly, all these characteristic bands associated with both BNNTs and silica are clearly present in the FTIR spectrum of the BNNT–silica composite microfibers, confirming the successful incorporation and structural integration of BNNTs within the silica matrix.

The XRD pattern of pure silica microfibers, shown in Figure 1e, exhibits a weak and broad diffraction peak centered at 2*θ* ≈ 22°, which is characteristic of scattering from nanocrystalline domains embedded within an otherwise amorphous silica matrix [29]. In contrast, the XRD pattern of BNNTs displays distinct characteristic diffraction peaks [30], most notably the (002) reflection located at 2*θ* ≈ 26.8°, corresponding to the interplanar spacing of the hexagonal BN lattice. For 0.5 wt.% BNNT–silica microfibers, no discernible BNNT diffraction peaks are observed. This absence is likely due to the low BNNT concentration and their uniform dispersion throughout the silica matrix, the latter being supported by the Raman mapping data shown in Supplementary Figure S2. Together, these factors reduce the overall nanotube scattering intensity below the instrument’s detection limit.

Figure 1f presents the thermogravimetric (TG) and corresponding differential thermogravimetric (DTG) curves, illustrating the thermal-treatment-induced weight-loss behavior of the microfibers. The DTG profile exhibits two distinct endothermic peaks at approximately 64 °C and 346 °C. The first peak corresponds to the evaporation of residual solvent trapped within the microfibers, while the second peak is attributed to the thermal degradation of the polymeric component (PEO) [31]. Above 400 °C, the TG curve shows a relatively stable plateau, indicating that the silica network remains largely intact and that the inorganic matrix undergoes minimal mass loss.

### 3.2. Mechanical Properties

**Figure 2:**
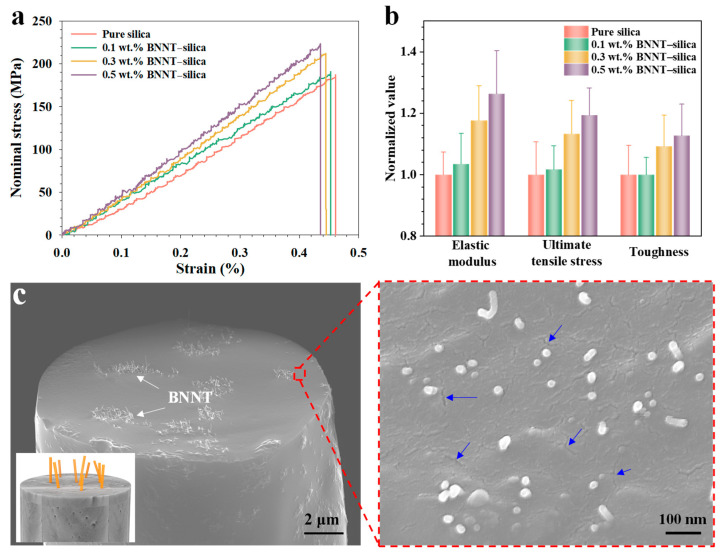
Mechanical characterization of electrospun BNNT–silica microfibers. (**a**) Representative stress–strain curves. (**b**) Comparison of the bulk mechanical properties on a normalized basis. (**c**) Representative SEM image showing protruding BNNTs from a fractured 0.5 wt.% BNNT–silica microfiber. The inset schematic in the bottom-left illustrates nanotube protrusion. The zoomed-in SEM image highlights small cracks on the silica fiber cross-section (marked by blue arrows).

Figure 2a presents representative stress–strain curves, all of which exhibit linear-elastic behavior up to fracture. The absence of yielding confirms the brittle nature of both pure silica and BNNT–silica microfibers. The incorporation of BNNTs into the silica matrix leads to significant enhancements in both Young’s modulus and tensile strength, with the degree of improvement increasing with BNNT content, as illustrated in Figure 2b and summarized in Table 1. Pure silica microfibers exhibit a Young’s modulus of ~40.6 GPa and a tensile strength of ~187.1 MPa. With the addition of 0.5 wt.% BNNTs, the Young’s modulus increases by 26.4% to 51.3 GPa, and the tensile strength rises by 19.4% to 223.3 MPa.

As the BNNT concentration increases, the fracture strain of the microfibers exhibits a slight reduction—from approximately 0.46% (pure silica) to 0.44% (0.5 wt.% BNNT)—indicating marginally reduced ductility. Nevertheless, the toughness of the microfibers increases with BNNT content. The unreinforced silica microfibers possess an average toughness of 430.9 kJ/m^3^, whereas the 0.5 wt.% BNNT–silica microfibers exhibit a 12.8% increase, reaching 486.0 kJ/m^3^. This enhanced energy-absorption capability is primarily attributed to the additional strain energy dissipation enabled by the BNNT reinforcements prior to the failure of the silica matrix. The results collectively highlight the crucial role of BNNTs in enhancing the stiffness, strength, and overall resilience of electrospun silica microfibers through effective load transfer and nanoscale reinforcement. A comparison of the bulk mechanical properties of BNNT-reinforced ceramic nanocomposites in the literature is shown in Table 2.

SEM imaging of the fractured cross-sectional surfaces of an individual microfiber, as shown in Figure 2c, reveals the failure mode of the nanocomposite microfiber. The fracture surface displays protruding nanotubes, resulting from their pullout from the silica matrix during tensile testing, which indicates effective interfacial load transfer between the nanotubes and the matrix. The frictional sliding of nanotubes within the matrix during pullout dissipates energy, thereby enhancing the material’s resilience. Overall, the protruding nanotubes exhibit uniform dispersion, with exposed lengths ranging from approximately 100 nm to 1 μm. Moreover, the nanotubes exhibit pronounced uniaxial alignment along the longitudinal axis of the silica microfiber, indicating favorable nanotube alignment. This observation aligns with prior reports of strong nanotube alignment in electrospun polymer nanocomposites [20,21,32]. During electrospinning, the viscous preceramic solution experiences electrostatic forces, and frictional interactions between the fluid and BNNTs promote their alignment within the matrix. The cross-section of the electrospun BNNT–silica microfiber exhibits numerous nanoscale cracks, with lengths extending to several hundred nanometers, as shown in the zoomed-in image in Figure 2c. This observation aligns with the density measurements, which further confirm the porous nature of the electrospun BNNT–silica microfibers.

**Table 2:** Comparison of the bulk mechanical properties of BNNT-reinforced ceramic nanocomposites.

Matrix Material	Manufacturing Method	BNNT Concentration (wt.%)	Strength		Toughness		Reference
			Flexural Strength (MPa)	Tensile Strength (MPa)	Fracture Toughness (MPa·m^1/2^)	Toughness (kJ/m^3^)	
SiO_2_	Electrospinning	0	-	187.1	-	430.9	This work
		0.5	-	223.3	-	486.0	
	molding	0	52.2	-	0.58	-	[12]
		5	120.5	-	1.21	-	
	molding	0	27.3	-	0.58	-	[33]
		5	44.2	-	0.68	-	
	Additive manufacturing	0	13.7	-	-	-	[14]
		0.1	21.2	-	-	-	
	molding	0	23.0	-	0.54	-	[15]
		0.5	68.3	-	1.44	-	
	Additive manufacturing	0	14.0	-	0.20	-	[19]
		0.4	33.6	-	0.50	-	
Al_2_O_3_	Plasma spray coating	0	-	-	2.05	-	[13]
		5	-	-	3.10	-	
	molding	0	319	-	4.9	-	[34]
		2	532	-	6.1	-	
	molding	0	365.6	-	5.2	-	[35]
		1.5	580.9	-	6.1	-	
	Additive manufacturing	0	47.2	-	1.0	-	[16]
		0.6	80.6	-	1.8	-	
ZrO_2_	molding	0	895.5	-	7.94	-	[36]
		1	1143.3	-	13.13	-	
Si_3_N_4_	molding	0	895	-	7.1	-	[37]
		1.5	1150	-	8.2	-	
SiOC	molding	0	55.5	-	0.9	-	[17]
		1.0	137.4	-	3.0	-	

### 3.3. Nanotube Alignment Inside the Matrix

**Figure 3:**
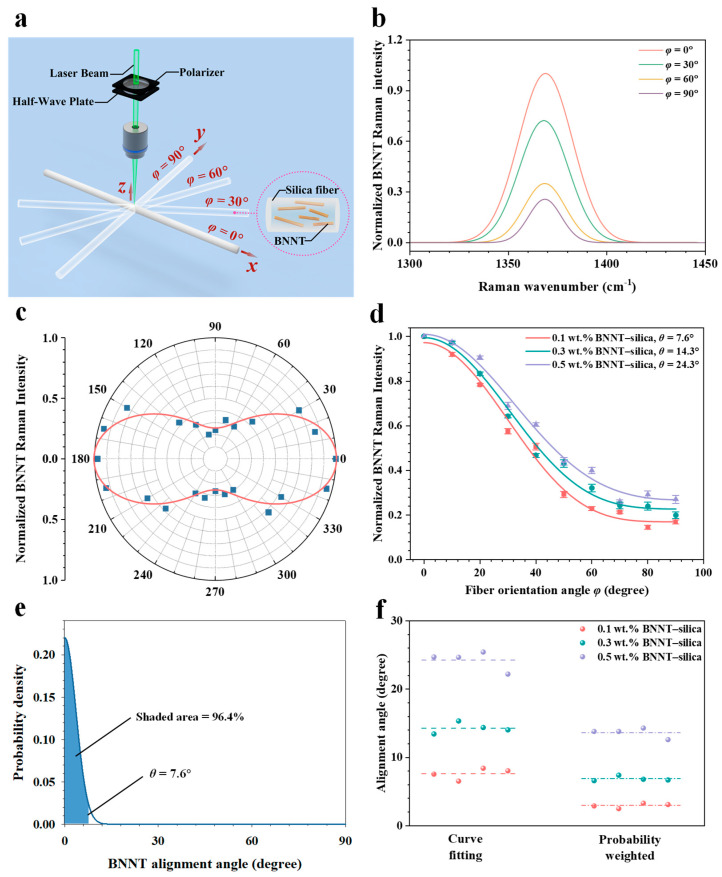
Polarized Raman measurements of nanotube alignment within BNNT–silica microfibers. (**a**) Schematic of the experimental setup. (**b**) Raman G-band spectra of 0.5 wt.% BNNT–silica microfibers at selected microfiber orientation angles. (**c**) Angular dependence of the BNNT G-band peak intensity. (**d**) Variation of the BNNT G-band peak intensity with microfiber orientation angle and corresponding fitted curve based on Equation (2). (**e**) Predicted BNNT alignment angle distribution based on the Gaussian distribution model for the tested 0.1 wt.% BNNT–silica microfiber. (**f**) Comparison of BNNT alignment angles at various nanotube concentrations obtained through curve fitting and probability-weighting analysis.

Nanotube alignment is a crucial factor influencing the bulk mechanical properties of nanocomposites. In this study, the collective alignment of BNNTs within silica microfibers is quantitatively examined using polarized Raman spectroscopy. Owing to the one-dimensional structural nature of nanotubes, their Raman scattering intensity is highly sensitive to orientation. Figure 3a shows the schematic of the vertical/vertical (VV) polarized Raman measurement setup. The incident laser beam, propagating along the *z*-axis, is polarized within the *xz*-plane using a linear polarizer, followed by a half-wave plate with its fast axis oriented at 45° relative to the polarizer’s transmission axis.

Figure 3b presents selected polarized Raman spectra from 0.5 wt.% BNNT–silica microfibers, showing a well-defined G-band centered at approximately 1369 cm^−1^. The G-band peak intensity decreases monotonically as the microfiber orientation angle increases from 0° to 90°. This orientation-dependent variation in Raman intensity indicates anisotropic alignment of BNNTs within the silica matrix. Figure 3c shows a polar plot of the variation in BNNT G-band intensity with rotation angle, ranging from 0° to 360°. The degree of collective nanotube alignment is quantified from the polarized Raman measurements using the model proposed by Gorman et al. [38], wherein the *VV*-polarized Raman scattering intensity as a function of nanotube orientation angle is expressed as

(2) $$\mathrm{VV}\;(\varphi )\;\propto \;p\int_{\varphi -\theta }^{\varphi +\theta }\cos^{4}(\varphi )d\varphi +(1-p)\int_{\varphi +\theta }^{\pi +\varphi -\theta }\cos^{4}(\varphi )d\varphi$$

where *φ* denotes the rotational angle of the microfiber relative to the polarization direction of the excitation laser, *θ* represents the alignment angle between the nanotube’s longitudinal axis and the microfiber’s lengthwise axis, and *p* denotes the mole fraction of nanotubes oriented within ±*θ* of the microfiber’s longitudinal axis. The remaining fraction, 1 *− p*, corresponds to nanotubes distributed over orientation angles from *θ* to π/2 and from −*θ* to −π/2.

Figure 3d presents the curve fitting of selected polarized Raman measurement data using Equation (2). Microfibers containing 0.1 wt.% BNNTs exhibit the steepest decline in G-band intensity as the rotation angle *φ* increases from 0° to 90°, corresponding to an overall nanotube alignment of *θ* = 7.6° and *p* = 96.2%. This indicates that approximately 96.2% of the nanotubes are oriented within 7.6° of the microfiber’s longitudinal axis. In comparison, the *θ* and *p* values for microfibers with 0.3% and 0.5% BNNT loadings are 14.3° and 90.4%, and 24.2° and 84.5%, respectively, which are summarized in Table 3. These results demonstrate better nanotube alignment at lower BNNT loadings. This trend can be attributed to the larger inter-nanotube spacing and, consequently, weaker inter-nanotube interactions, which facilitate better nanotube dispersion within the ceramic matrix. Excellent nanotube alignment enhances interfacial load transfer, thereby increasing the load carried by the nanotubes and contributing to the effective mechanical reinforcement of the ceramic composite.

It is worth noting that the nanotube alignment data obtained from polarized Raman spectroscopy represent collective measurements. To analyze interfacial stress-transfer properties using these data, assumptions regarding the nanotube orientation distribution must be made. In this study, the orientations of all nanotubes within the microfibers are assumed to follow a normal distribution within the angular range of 0° to 90°. Figure 3e illustrates the nanotube orientation distribution profile for the 0.1 wt.% BNNT–silica microfiber. Based on the normal distribution model, the probability-weighted mean nanotube alignment angle is approximately 3.0°. Similarly, the probability-weighted mean nanotube alignment angles for the 0.3 wt.% and 0.5 wt.% BNNT–silica microfibers are estimated to be about 6.9° and 13.6°, respectively, as shown in Figure 3f.

### 3.4. In Situ Raman Micromechanical Measurements

**Figure 4:**
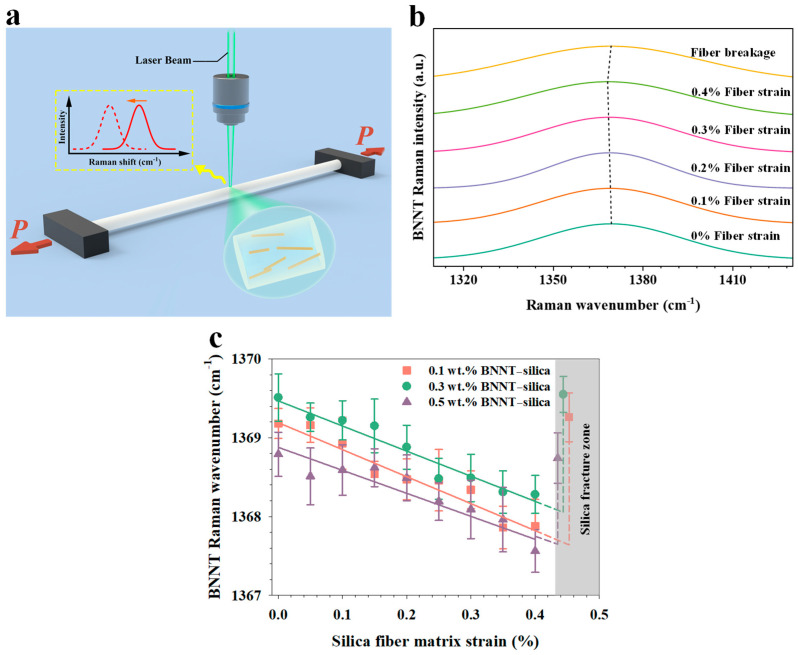
In situ Raman micromechanical measurements of electrospun BNNT–silica microfibers. (**a**) Schematic of the experimental setup. (**b**) Selected Raman spectra of a 0.5 wt.% BNNT–silica microfiber under varying tensile strains. (**c**) Dependence of the BNNT G-band shift on applied tensile strain to the silica matrix.

We conducted in situ Raman micromechanical measurements, as illustrated in Figure 4a, to characterize interfacial load transfer in BNNT–silica microfibers. Figure 4b presents representative Raman spectra of the BNNT G-band for a 0.5 wt.% BNNT–silica microfiber subjected to progressively increasing tensile strains up to fracture. The Raman wavenumber of the characteristic BNNT G-band gradually shifts to lower values as tensile strain is applied. This strain-dependent Raman shift arises from the elongation of B–N bonds [39] and is fully reversible up to the point of microfiber fracture. These results indicate that, prior to fracture, the BNNT–silica interface remains intact under applied loading, in contrast to the interfacial slippage observed in electrospun BNNT-reinforced polymer microfibers [21]. This behavior can be attributed to the strong interfacial binding between BNNTs and the silica matrix, as well as the relatively low fracture strain of the ceramic matrix. The strain-induced Raman shift disappears once the microfiber fractures.

Figure 4c presents the measured Raman wavenumber of the BNNT G-band as a function of the applied tensile strain to the silica matrix. The Raman wavenumber decreases approximately linearly with increasing tensile strain. Curve fitting yields average Raman downshift rates of about 3.4 cm^−1^/%, 3.2 cm^−1^/%, and 2.9 cm^−1^/% for BNNT loadings of 0.1 wt.%, 0.3 wt.%, and 0.5 wt.%, respectively. A higher downshift rate indicates better nanotube alignment within the silica matrix, consistent with polarized Raman measurements. Notably, these downshift rates exceed those reported for BNNT–silica nanocomposites produced via digital light processing techniques (~2.7 cm^−1^/%), in which nanotubes are randomly oriented [19]. This observation aligns with prior reports [40] and demonstrates that improved nanotube alignment enhances interfacial load transfer and, consequently, the mechanical reinforcement efficiency of the nanotubes.

### 3.5. Interfacial Load Transfer Characteristics

**Figure 5:**
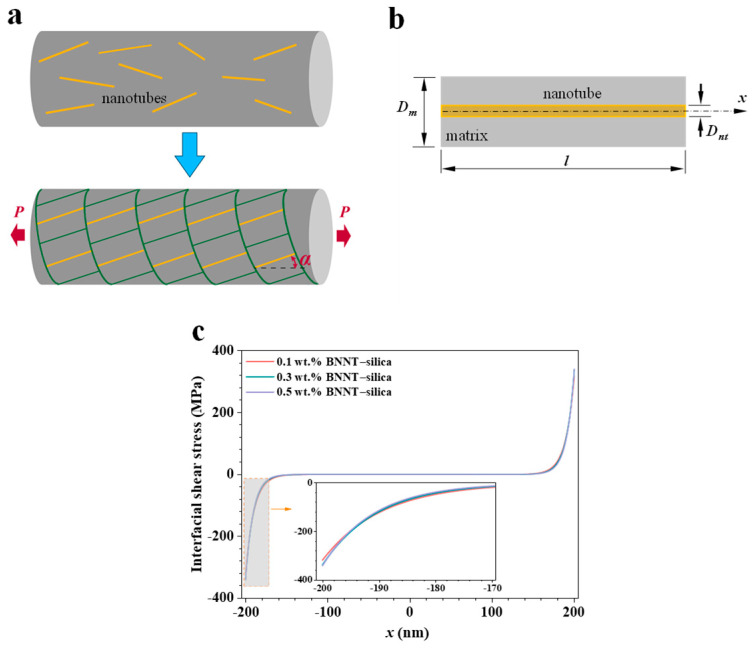
Interfacial load transfer characteristics of electrospun BNNT–silica microfibers. (**a**) Schematics of a nanotube-reinforced composite fiber (**top**) and its simplified equivalent configuration (**bottom**). (**b**) Schematic of a single-nanotube nanocomposite. (**c**) Calculated interfacial shear stress distribution profiles at the respective failure strains for embedded nanotubes with a length of 400 nm.

Polarized Raman spectroscopic analysis reveals a polydisperse orientation distribution of nanotube reinforcements within the microfiber, as illustrated in the upper portion of Figure 5a. To elucidate the local interfacial stress-transfer characteristics of BNNT–silica microfibers, an equivalent nanotube–matrix composite model is developed. In this model, all nanotube fillers are assumed to be straight and possess identical diameters, lengths, and orientation angles (α) relative to the direction of the applied uniaxial force, as schematically depicted in the lower portion of Figure 5a. Figure 5b shows the configuration of an equivalent single-nanotube composite, where a nanotube of diameter $D_{\mathrm{nt}}\;$ and length  l  is concentrically embedded within a cylindrical silica matrix of the same length and an external diameter $D_{m}$. Given the low BNNT loading fractions, inter-nanotube interactions are considered negligible. The pointwise interfacial shear stress (IFSS) distribution along the BNNT–silica interface can be described using a shear–lag micromechanics model [20,21], given as

(3) $$\tau =\frac{nE_{\mathrm{nt}\;}\epsilon \cos^{2}\alpha }{2}\frac{\cosh\left(\frac{2\mathrm{nx}}{D_{\mathrm{nt}}}\right)}{\sinh\left(\frac{\mathrm{nl}}{D_{\mathrm{nt}}}\right)}$$

where *x* is the coordinate along the longitudinal axis of the nanotube, *ϵ* represents the external uniaxial strain imposed on the matrix, and n is defined as $n=\sqrt{\frac{E_{m}}{E_{\mathrm{nt}\;}(1+v_{m})\cdot \log\left(D_{m}/D_{\mathrm{nt}}\right)}}$, in which $E_{m}$ and $v_{m}$ are the Young’s modulus and Poisson’s ratio of the matrix, respectively, and $E_{\mathrm{nt}}$ is the Young’s modulus of BNNTs. Under the uniaxial failure strain $\epsilon_{\mathrm{cr}}$, the maximum IFSS along the BNNT–silica interface occurs at the two ends of the nanotube (i.e., *x* = ±*l*/2), and is represented as:

(4) $$\tau_{\max\;}=\;\frac{nE_{\mathrm{nt}\;}\epsilon_{\mathrm{cr}}\cos^{2}\alpha }{2\tanh\left(\frac{\mathrm{nl}}{D_{\mathrm{nt}}}\right)}$$

It is noted that the interfacial shear stress is strongly correlated with the nanotube alignment angle, as quantified in Equations (3) and (4).

Figure 5c presents the pointwise IFSS distribution profiles along the BNNT–silica interface with different BNNT loadings upon failure. The calculated equivalent $\tau_{\max}$ values are approximately 318.0 MPa (0.1 wt.%), 337.9 MPa (0.3 wt.%), and 340.8 MPa (0.5 wt.%). These calculations are based on the following parameters: $E_{\mathrm{nt}}$ = 1070 GPa [41]; $E_{m}$ = 70 GPa and $v_{m}$ = 0.17 [42]; $D_{\mathrm{nt}}$ = 2.9 nm (median BNNT diameter) [23]; l = 400 nm; $D_{m}$ calculated to be approximately 70 nm (0.1 wt.%), 41 nm (0.3 wt.%), and 31 nm (0.5 wt.%), based on the densities of silica (2.3 g/cm^3^) [26] and BNNTs (1.35 g/cm^3^) [21]. Effective stress transfer occurs only within a limited region near each nanotube end. Assuming an IFSS threshold of 1 MPa, the effective transfer length at each end is approximately 70 nm (0.1 wt.%), 64 nm (0.3 wt.%), and 62 nm (0.5 wt.%). The corresponding maximum axial (normal) stress within the BNNT occurs near the central region and is approximately 4.8 GPa (0.1 wt.%), 4.6 GPa (0.3 wt.%), and 4.4 GPa (0.5 wt.%), all of which are well below the tensile strength of BNNTs.

It is noteworthy that the maximum IFSS value obtained for the electrospun BNNT–silica microfibers is substantially lower than that reported from single-nanotube pullout experiments (1252 MPa [11]), yet significantly higher than those observed in conventionally molded BNNT–silica (92 MPa [15]) and additively manufactured BNNT–silica (136 MPa [19]) nanocomposites. Single–nanotube pullout measurements are conducted on individual nanotubes partially embedded in engineered ceramic/nanotube/ceramic thin-film structures. The exceptionally strong BNNT–silica interfacial binding in such systems is enabled by the ultra-high strength of BNNTs and reflects the intrinsic binding interaction between individual, straight nanotubes and the surrounding silica matrix, effectively eliminating the influence of common structural imperfections found in bulk composites—such as nanotube bundling, entanglement, and waviness. In contrast, nanotubes in molded and additively manufactured silica composites are randomly oriented and exhibit pronounced structural nonidealities that weaken their interfacial bonding with the ceramic matrix. Moreover, the maximum IFSS in these bulk BNNT–silica composites is limited by the inherent brittleness of the matrix, rather than the interfacial strength. Therefore, the interfacial load transfer capacity and the reinforcement efficiency of BNNTs could be further increased with the improvement of the matrix’s failure strain.

## 4. Conclusions

This study demonstrates the effectiveness of BNNTs as nanoscale reinforcements for electrospun silica microfibers, highlighting their exceptional potential to enhance the mechanical performance of ceramic composites. The substantial improvements in bulk mechanical properties with small BNNT loadings are primarily attributed to the excellent nanotube alignment achieved through the electrospinning process and the effective interfacial load transfer at the nanotube–ceramic interface. The findings highlight the crucial role of processing-induced nanotube alignment and interfacial mechanics in determining the macroscopic behavior of ceramic nanocomposites. The synergistic integration of BNNTs into the silica matrix via electrospinning provides a model system for quantitatively studying nanoscale load transfer in one-dimensional nanofiller-reinforced ceramics. Overall, this work advances understanding of the process–structure–property relationship governing the mechanical performance of nanotube-reinforced ceramics. It offers valuable insights for the rational design and scalable manufacturing of next-generation lightweight, strong, tough, and thermally stable ceramic materials, with promising implications for applications in aerospace, electronics, and other demanding engineering environments.

## Figures and Tables

**Table 1:** Summary of the mechanical properties of BNNT–silica microfibers.

Property	0 wt.% BNNT	0.1 wt.% BNNT	0.3 wt.% BNNT	0.5 wt.% BNNT
Young’s Modulus (GPa)	40.6 ± 3.0	42.0 ± 4.0	47.8 ± 4.6	51.3 ± 5.7
Tensile Strength (MPa)	187.1 ± 20.2	190.4 ± 14.4	212.0 ± 20.1	223.3 ± 16.5
Toughness (kJ/m^3^)	430.9 ± 41.2	431.1 ± 24.0	470.7 ± 43.7	486.0 ± 43.9
Breaking Strain (‰)	4.6 ± 0.6	4.5 ± 0.5	4.4 ± 0.4	4.4 ± 0.4
Maximum IFSS (MPa)	-	318.0 ± 34.4	337.9 ± 32.0	340.8 ± 33.7

**Table 3:** Summary of nanotube alignment within electrospun BNNT–silica microfibers based on polarized Raman measurements.

	0.1 wt.% BNNT			0.3 wt.% BNNT			0.5 wt.% BNNT		
Sample No.	*p* (%)	*θ* (°)	Probability-Weighted Average Angle (°)	*p* (%)	*θ* (°)	Probability-Weighted Average Angle (°)	*p* (%)	*θ* (°)	Probability-Weighted Average Angle (°)
1	96.4	7.6	2.9	89.7	13.4	6.6	84.8	24.7	13.8
2	96.6	6.5	2.5	90.2	15.3	7.4	84.6	24.7	13.8
3	95.8	8.4	3.3	91.0	14.4	6.8	84.5	25.4	14.3
4	95.9	8.0	3.1	90.7	14.0	6.7	83.9	22.2	12.6
Average and deviation	-	7.6 ± 0.8	3.0 ± 0.3	-	14.3 ± 0.8	6.9 ± 0.4	-	24.2 ± 1.4	13.6 ± 0.7

## Data Availability

The original contributions presented in this study are included in the article/Supplementary Material. Further inquiries can be directed to the corresponding author.

## Supplementary Materials

The following supporting information can be downloaded at: https://www.mdpi.com/article/10.3390/nano16010069/s1, Figure S1: The diameter distribution profile of the tested BNNT–silica microfibers; Figure S2: Raman surface mapping of the BNNT G-band peak frequency for a 0.5 wt.% BNNT–silica microfiber, overlaid on the corresponding optical image.
