# Supplementary material for: Mechanical Enhancements of Electrospun Silica Microfibers with Boron Nitride Nanotubes
Source: Nanomaterials (Basel). 2026 Jan 3;16(1):69. doi: 10.3390/nano16010069 (PMC12787889; doi:10.3390/nano16010069)
Supplement: Supplementary file 1 [file nanomaterials-16-00069-s001.zip › nanomaterials-4037469-supplementary.pdf]

Supplementary Material

Mechanical Enhancements of Electrospun Silica Microfibers with Boron Nitride Nanotubes

Dingli Wang [1], Nasim Anjum [1], Zihan Liu [1] and Changhong Ke [1,2,*]

[1] Department of Mechanical Engineering, State University of New York at Binghamton, Binghamton, NY, 13902, USA; dwang86@binghamton.edu (D.W.); nanjum3@binghamton.edu (N.A.); zliu154@binghamton.edu (Z.L.)
[2] Materials Science and Engineering Program, State University of New York at Binghamton, Binghamton, NY, 13902, USA
* Correspondence: cke@binghamton.edu

Supplementary Material Captions:

1. Supplementary Fig. S1

2. Supplementary Fig. S2

1. Supplementary Fig. S1

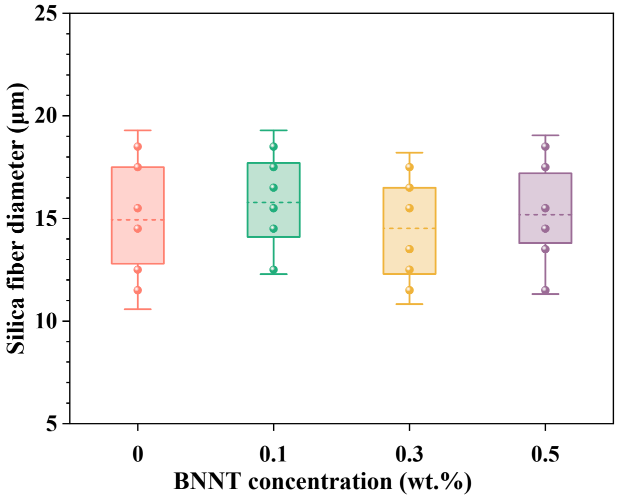

Figure S1. The diameter distribution profile of the tested BNNT–silica microfibers.

2. Supplementary Fig. S2

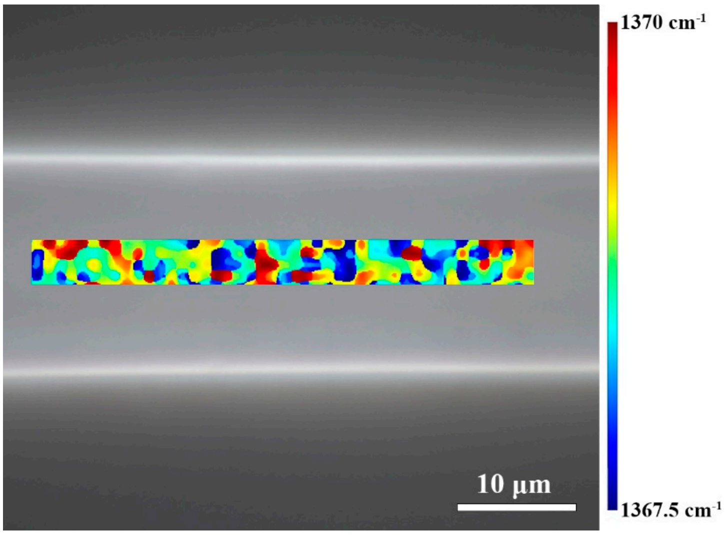

Figure S2. Raman surface mapping of the BNNT G-band peak frequency for a 0.5 wt.% BNNT–silica microfiber, overlaid on the corresponding optical image. The mapped area is 3.6 × 42 μm^2, with a step size equal to the laser spot diameter (1.2 µm). The characteristic BNNT G-band is consistently observed at each Raman measurement location, with the peak frequency indicated by the color scale. These data support a uniform dispersion of BNNTs throughout the silica matrix.
